# Arginine Enhances Ovarian Antioxidant Capability via Nrf2/Keap1 Pathway during the Luteal Phase in Ewes

**DOI:** 10.3390/ani12162017

**Published:** 2022-08-09

**Authors:** Yan Ma, Zhiyi Guo, Qiujue Wu, Binyao Cheng, Zhenhan Zhai, Yuqin Wang

**Affiliations:** College of Animal Science and Technology, Henan University of Science and Technology, Luoyang 471003, China

**Keywords:** arginine, ovarian antioxidant system, malnutrition, Nrf2/Keap1 signal pathway, sheep

## Abstract

**Simple Summary:**

The quantity and quality of forage grass may decrease to some extent in autumn and winter, and feeding with such forage grass may temporarily cause malnutrition and ovarian oxidative stress in ewes. Arginine is one of the most abundant amino acids enhancing the antioxidant capability in animal tissues. Thus, this study investigated the effects of arginine on ovarian antioxidant capability during the luteal phase in ewes. Our results showed that nutrition restriction can reduce the ovarian antioxidant capability in ewes, while arginine supplementation can significantly improve it, which was associated with the nuclear factor erythroid 2-related factor 2/Kelch-like ECH-associated protein 1 pathway.

**Abstract:**

This study evaluated the effect of arginine (Arg) on ovarian antioxidant capability during the luteal phase in ewes. A total of 108 multiparous Hu sheep at two years of age were randomly allocated to three groups: a control group (CG), a restriction group (RG), and an Arg group (AG), with six replicates per group and six ewes per replicate. Our results showed that the end body weight was significantly decreased in the RG group (*p* < 0.05), while the Arg addition reversed this reduction. The estrous cycle days were significantly increased in the RG group (*p* < 0.05), while Arg addition reversed this time extension. Compared with the control group, restricting feeding could significantly enhance the number of small follicles (SF), total follicles (TF), large corpora lutea, and the SF/TF (*p* < 0.05), while Arg addition reduced the number of SF and TF. However, the large follicles/TF were significantly decreased (*p* < 0.05), while Arg addition reversed this reduction. In addition, nutrition restriction significantly increased the malondialdehyde (MDA) level (*p* < 0.05), while significantly decreased the glutathione/glutathione disulfide and the activities of superoxidative dismutase, catalase, and glutathione peroxidase in the ovaries (*p* < 0.05). However, Arg addition reversed this enhancement of the MDA level and the reductions in these antioxidant enzymes activities. In addition, positive relationships occurred between antioxidant enzyme activities and the enzyme mRNA expressions. Meanwhile, the nuclear factor erythroid 2-related factor 2 (Nrf2) mRNA expression was positively connected with antioxidant mRNA expressions and negatively related to the Kelch-like ECH-associated protein 1 (Keap1) mRNA expression. The Nrf2 protein expression was negatively related to the Keap1 protein expression. In conclusion, nutrition restriction reduced the ovarian antioxidant capability in ewes, while this was significantly improved by Arg supplementation, which was associated with the Nrf2/Keap1 pathway.

## 1. Introduction

Nutrition levels in feedstuff have important effects on metabolic function and can affect the reproductive performance in ruminants [1]. Malnutrition can lead to delayed estrus in ewes, disrupted estrus cycles, a reduced pregnancy rate, and a low fetal birth weight [1,2]. The periodic follicular development in sexually mature female sheep is closely related to the nutrition levels in the luteal phase. Malnutrition in the luteal phase can affect the fecundity by inhibiting follicular development, delaying estrus, changing the microenvironmental homeostasis in the follicles, and increasing the proportion of atresia follicles [3]. Short-term nutritional supplementation may maintain the homeostasis of the microenvironment in follicles and promote follicular development during the luteal phase [4].

The quantity and quality of forage grass may decrease to some extent in autumn and winter, and feeding with such forage grass may temporarily cause malnutrition and ovarian oxidative stress in mammals [5]. When the ovarian antioxidant system’s calm is broken, intracellular reactive oxygen species (ROS) assemble, which are supervised by malondialdehyde (MDA). Glutathione (GSH) and glutathione disulfide (GSSG), as the pivotal components in the intracellular non-enzyme system, play a key role in maintaining the stability of the antioxidant system [6]. In addition, the antioxidant enzyme’s activities might be reduced during the luteal phase, such as that of catalase (CAT), glutathione peroxidase (GSH-Px), and superoxidative dismutase (SOD). At the translation level, the enzyme activities can be regulated by the upstream antioxidant enzyme genes [7]. As we know, signaling pathways regulate the gene expressions of antioxidant enzymes. Nuclear factor erythroid 2-related factor 2 (Nrf2) is one of the important regulation factors of the intracellular antioxidant system. Nrf2 is an inactive complex, bound to a suppressive molecule known as Kelch-like ECH-associated protein 1 (Keap1) when the cell is in a stable state [8,9]. Nevertheless, when the antioxidant system is affected, Nrf2 will be released from Keap1 in the cytoplasm and will regulate the downstream gene expressions of antioxidant enzymes. 

Arginine (Arg) is one of the most abundant amino acids in animal tissues. Convincing evidence has been presented that Arg plays an important role in enhancing the antioxidant system in mammals [10]. Previous studies have reported that Arg can inhibit oxidative stress and enhance antioxidant capacity via the Nrf2/Keap1 pathway in rats [2,10]. Thus, we speculated that Arg addition might enhance the antioxidant capability via the Nrf2/Keap1 pathway in ovaries, which is studied in this research. This study investigated the effect of Arg on ovarian antioxidant capability during the luteal phase in ewes. Furthermore, the gene expressions of antioxidant enzymes and the Nrf2/Keap1 pathway were investigated to uncover the molecular mechanism of Arg on the enhancement of the antioxidant ability in the ovaries of ewes.

## 2. Materials and Methods

### 2.1. Ethical Standards

All procedures in this study were approved by the Animal Welfare Committee of Henan University of Science and Technology, P.R. China, and the national guidelines for the care and use of animals (approve number: 094-2022).

### 2.2. Animals and Treatments

A total of 108 multiparous Hu sheep of two years were raised on the same farm in Luoyang, China. The estrus synchronization for all ewes was performed by inserting intravaginal progestogen sponges for 12 days followed by intramuscular administration of prostaglandin F2α. All ewes were monitored for estrous behavior at 8:30 h and 16:30 h after pessary removal. The observed end of estrous behavior was designed as day 0 of the estrous cycle. On day 6, the ewes were randomly divided into three groups: the control group, the restriction group, and the Arg group. Each group had six replicates, and each replicate had six ewes. All ewes were provided with a total mixed ration diet. The composition of the basal diet and nutrient content are shown in Table 1. The ewes in the control group received a 1× maintenance diet (1040 g/d), and the ewes in the restriction group and Arg group received a 0.5× maintenance diet (520 g/d). The body weight (BW) was determined at the beginning and the end of the feeding experiment. From day 6 of the estrous cycle to the end of the experiment, the animals in the control group and restriction group were injected with 5 mL of sterile saline solution intravenously, while the Arg group was injected with 5 mL of L-Arg-HCL solution (155 mmol Arg/kg BW) 3 times daily. The concentration of Arg was referenced in a previous study [2]. The estrous behaviors of all ewes were detected from day 15 of the cycle, and the ewes were euthanized on the second day after estrous behavior resumed. 

### 2.3. Collection of Samples

After being anesthetized, all ewes in each group were euthanized by cutting the jugular vein. The blood samples were collected and sent to the laboratory for the further analysis. Thereafter, the abdominal cavity of each ewe was dissected, and the ovaries were taken out and stored in liquid nitrogen for further experiments. Both visible follicles (>1.0 mm) and corpora lutea (CL) were dissected and collected from the ovarian stroma and placed in a sterile dish for further measurements.

### 2.4. Numbers of Follicles and Corpora Lutea

The ovaries and carcass were weighed by using a precision balance. The numbers of follicles and corpus luteum were counted visually. The follicles were divided into three groups, including small follicles (SF, diameter ≤ 2 mm), middle-sized follicles (MF, 2 mm < diameter < 5 mm) and large follicles (LF, diameter ≥ 5 mm). Similarly, the corpus luteum were divided into two groups, including small CL (SCL, diameter < 5 mm) and large CL (LCL, diameter ≥ 5 mm). The diameters of the follicles and CL were determined by a high-precision digital display micrometer.

### 2.5. Serum Profiles Analysis

The concentrations of Arg, ornithine (Orn), citrulline (Cit), glutamic acid (Glu), and proline (Pro) in the serum of ewes were determined by an ELISA kit (NeoBioscience, Shenzhen, China) following the manufacture’s protocol. In short, we added the standard solution of different concentrations at 50 µL to the standard wells, and we added the 10 µL sample solution and 40 µL diluent to the sample wells. Then, we added 100 µL of the HRP-labeled detection antibody to each well, sealed the reaction well with a sealing plate membrane, and immersed the well in a 37 °C water bath for 60 min. After adding the substrate and stop solution into each well, the OD value in each well was detected at 450 nm using an enzyme standard instrument.

### 2.6. Sex Hormone Assay

Sex hormone levels in the serum of ewes were measured by ELISA analysis kits (NeoBioscience, Shenzhen, China) following the manufacturer’s instructions, including estradiol (E2) and progesterone (P4). In short, after the reagents were equilibrated at room temperature (18–25 °C) for 30 min, the concentrated washing solution was diluted 1:20 with distilled water. We added the standard solution of different concentrations at 50 µL to the standard wells, and we added the 10 µL sample solution and 40 µL diluent to the sample wells. Then, we added 100 µL of the HRP-labeled detection antibody to each well, sealed the reaction well with a sealing plate membrane, and immersed it in a 37 °C water bath for 60 min. After adding the substrate and stop solution into each well, the OD value in each well was detected at 450 nm using an enzyme standard instrument.

### 2.7. Determination of Antioxidant Capacity Biomarkers

The total protein concentrations in the ovaries were determined by the bicinchoninic acid method using a total protein assay kit (NeoBioscience, Shenzhen, China). The levels of GSH, GSSG, MDA, and the activities of SOD, CAT, GR, and GSH-Px in the ovaries were measured using the respective analysis kits (NeoBioscience, Shenzhen, China) following the manufacturers’ protocols. 

### 2.8. Quantitative Real-Time PCR Analysis

The total RNA was isolated from the ovaries using an RNAiso Kit (NeoBioscience, Shenzhen, China). The quality and concentration of RNA were determined by denatured RNA electrophoresis. The samples of RNA purity identified to exhibiting an OD260/OD280 ratio between 1.8 and 2.0 were chosen for further experiments. Quantitative real-time PCR was performed containing the PCR Master Mix (NeoBioscience, Shenzhen, China), RNase free dH2O, cDNA, and forward and reverse primers, including Nrf2, Keap1, CAT, GR, GSH-Px, manganese superoxide dismutase (MnSOD), copper-zinc superoxide dismutase (CuZnSOD), and beta-actin (β-actin, housekeeping gene), which are presented in Table 2. The PCR procedure was as follows: 94.5 °C for 30 s followed by 42 cycles of 94.5 °C for 5 s, 60 °C for 10 s, and 71.5 °C for 35 s.

### 2.9. Western Blot Analyses

Ovarian Nrf2 and Keap1 proteins were detected using the Western blot analysis. The radioimmunoprecipitation method was used to extract the total proteins. The proteins were separated by electrophoresis on 10% polyacrylamide gels AND then transferred to polyvinylidene difluoride membranes (Givers Technology Co., Ltd., Suzhou, China). After incubation in blocking buffer with 5.5% skim milk for 21 h, the membranes were incubated overnight with Nrf2 (1:500, Absin Biotechnology, Shanghai, China), Keap1 (1:500, Absin Biotechnology, Shanghai, China), and β-actin (1:3000, Absin Biotechnology, Shanghai, China) antibodies. The secondary antibody against rabbit IgG was incubated with the membranes. The protein signals were detected using a Western blot detection system. The chemiluminescent intensity of Nrf2 and Keap1 was quantified by IMAGEJ software, and the relative abundance of each protein was expressed as the ratio of optical density.

### 2.10. Statistical Analyses

SPSS21.0 (SPSS Inc., Chicago, IL, USA) was used to analyze the one-way ANOVA. Tukey’s multiple range test was performed when there were different significances among any groups (*p* < 0.05). The effects of time and nutritional treatment on the amino acid metabolism in the serum of ewes were evaluated by two-way ANOVA. Pearson’s correlation was performed to analyze the correlations of the mRNA expressions. 

## 3. Results

### 3.1. Effects of Diet and Arginine on Body Weight, Ovarian Weight, and the Number of Follicles and Corpus Luteum in Ewes

Compared with the other groups, the estrous cycle days were significantly increased in the RG group (*p* < 0.05), while Arg addition reversed this time extension. There were no different significances in the starting body weight (SBW), ending body weight (EBW), ovarian weight, carcass weight, and ovary/EBW between the groups. Compared with the control group, restricting feeding significantly enhanced the number of SF and TF (*p* < 0.05), and the SF/TF (*p* < 0.05), while Arg addition partly reduced the number of SF and TF. However, the LF/TF was significantly decreased compared to the control group (*p* < 0.05), while Arg addition reversed this reduction. Compared with the other groups, the number of LCL was significantly increased in the AG group (*p* < 0.05; Table 3).

### 3.2. Effects of Diet and Arginine on the Amino Acid Metabolism in the Serum of Ewes

Compared with the control group, the levels of Glu, Cit, Pro, and Orn in the serum were significantly increased in the RG group (*p* < 0.05), while Arg addition partly reversed the enhancements of Pro and Orn levels in the ewes (*p* < 0.05). In addition, there was no significant difference from day 9 to day 13 (Table 4).

### 3.3. Effects of Diet and Arginine on the Sex Hormone Levels in the Follicular Fluid of Ewes

**Figure 1:**
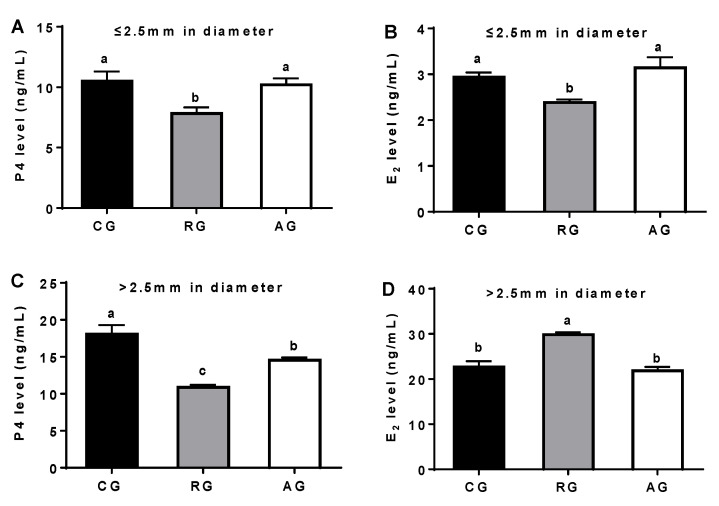
Effects of diet and arginine on sex hormone levels in follicular fluid of ewes. (**A**): P4 level in follicular fluid (follicular diameter ≤ 2.5 mm); (**B**): E_2_ level in follicular fluid (follicular diameter ≤ 2.5 mm); (**C**): P4 level in follicular fluid (follicular diameter > 2.5 mm); (**D**): E_2_ level in follicular fluid (follicular diameter > 2.5 mm). Abbreviations: CG, control group; RG, restriction group; AG, Arg group; P4, progesterone; E_2_, estradiol. Values are means ± SE (n = 6). Columns with different superscripts differ significantly among any groups (*p* < 0.05).

In the follicular fluid (follicular diameter ≤ 2.5 mm), the levels of P4 and E2 were significantly decreased in the RG group compared with the control group (*p* < 0.05), while Arg addition completely reversed these reductions. In the follicular fluid (follicular diameter > 2.5 mm), compared with the control group, the P4 level was significantly decreased in the RG group (*p* < 0.05), while Arg supplementation partly reversed this reduction. In contrast, the E_2_ level was significantly increased in the RG group (*p* < 0.05), while Arg addition completely reversed this enhancement (Figure 1).

### 3.4. Effects of Diet and Arginine on the Antioxidant Capability in the Ovaries of Ewes

**Figure 2:**
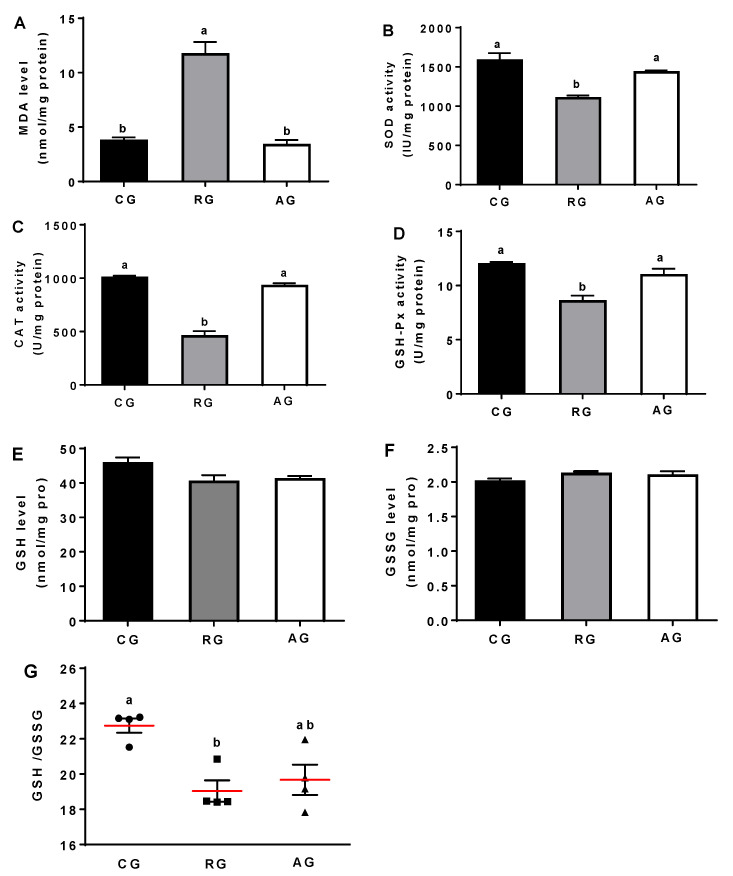
Effects of diet and arginine on the antioxidant capability in ovaries of ewes. (**A**): MDA level in ovary; (**B**): SOD activity in ovary; (**C**): CAT activity in ovary; (**D**): GSH-Px activity; (**E**): GSH level in ovary; (**F**): GSSH level in ovary; (**G**): GSH/GSSG. Abbreviations: CG, control group; RG, restriction group; AG, Arg group; MDA, malondialdehyde; SOD, superoxide dismutase; CAT, catalase; GSH-Px, glutathione peroxidase; GSH, glutathione; GSSG, oxidized glutathione. Values are means ± SE (n = 4). “▲,▪,•”means each measurement data. Columns with different superscripts differ significantly among any groups (*p* < 0.05).

Compared with the control group, the MDA level was significantly increased in the RG group (*p* < 0.05), while Arg addition completely reversed this enhancement. Conversely, the activities of SOD, CAT, and GSH-Px were significantly decreased in the RG group compared with the control group (*p* < 0.05), while Arg addition restored the activity of these antioxidant enzymes (*p* < 0.05). In addition, the levels of GSH and GSSG were not significantly different between the groups. However, the GSH/GSSG was significantly decreased in the RG group (*p* < 0.05), while Arg addition significantly enhanced this ratio (*p* < 0.05; Figure 2).

### 3.5. Effects of Diet and Arginine on the Antioxidant Enzyme Gene Expressions in the Ovaries of Ewes

**Figure 3:**
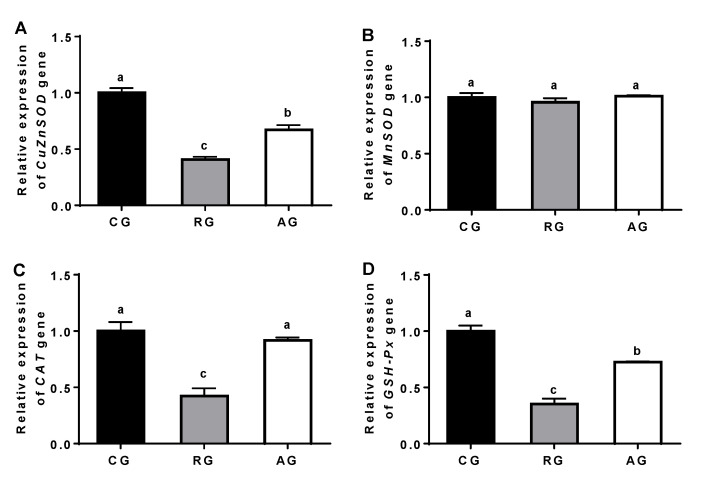
Effect of diet and arginine on antioxidant enzyme gene expressions in ovary of ewes. (**A**): Relative expression units of CuZnSOD gene; (**B**): Relative expression units of MnSOD gene; (**C**): Relative expression units of CAT gene; (**D**): Relative expression units of GSH-Px gene. Abbreviations: CG, control group; RG, restriction group; AG, Arg group; CuZnSOD, copper-zinc superoxide dismutase; MnSOD, manganese superoxide dismutase; CAT, catalase; GSH-Px, glutathione peroxidase. Values are means ± SE (n = 6). Columns with different superscripts differ significantly among any groups (*p* < 0.05).

Compared with the control group, the gene expressions of CuZnSOD, CAT and GSH-Px were significantly decreased in the RG group (*p* < 0.05), while Arg supplementation reversed these reductions (*p* < 0.05). In addition, the MnSOD gene expression was not significantly different between the groups (Figure 3). 

There were positive correlations between the mRNA levels of CuZnSOD (r = 0.843; *p* < 0.01), CAT (r = 0.798; *p* < 0.01), and GSH-Px (r = 0.803; *p* < 0.01) and the related enzymatic activities. In addition, positive correlations occurred between the Nrf2 mRNA expression and the mRNA levels of CuZnSOD (r = 0.794; *p* < 0.01), CAT (r = 0.743; *p* < 0.01), and GSH-Px (r = 0.813; *p* < 0.01) (Table 5).

### 3.6. Effects of Diet and Arginine on the Nrf2/Keap1 Pathway in the Ovaries of Ewes

**Figure 4:**
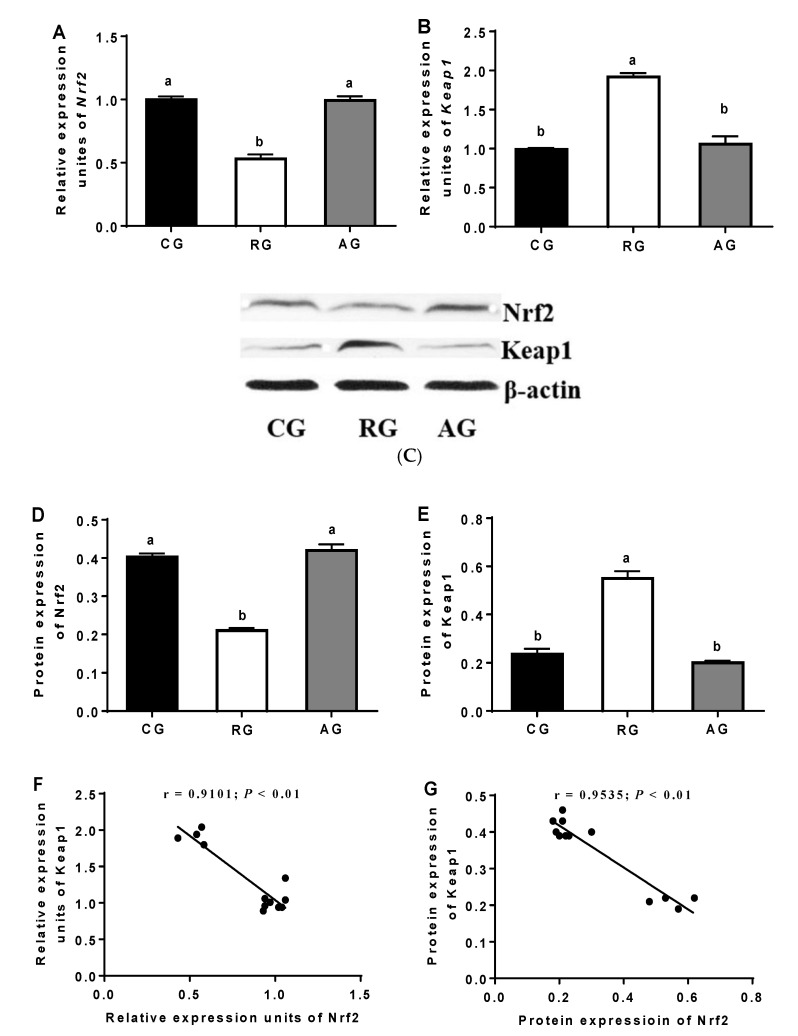
Effects of diet and arginine on Nrf2/Keap1 pathway in ovaries of ewes. (**A**): Relative expression units of Nrf2; (**B**): Relative expression units of Keap1; (**C**): The protein expressions of Nrf2 and Keap1 were determined by western blot; (**D**): Protein expression of Nrf2; (**E**): Protein expression of Keap1; (**F**): Pearson’s correlation analysis between Nrf2 and Keap1 mRNA levels; (**G**) Pearson’s correlation analysis between Nrf2 and Keap1 protein levels. Abbreviations: CG, control group; RG, restriction group; AG, Arg group; Nrf2, nuclear factor erythroid 2-related factor 2; Keap1, Kelch-like ECH-associated protein 1. Values are means ± SE (n = 4). Columns with different superscripts differ significantly among any groups (*p* < 0.05).

Compared with the control group, the expressions of the Nrf2 mRNA and protein were significantly decreased in the RG group (*p* < 0.05), while Arg addition completely reversed these reductions. In contrast, compared with the control group, the expressions of the Keap1 mRNA and protein were significantly increased in the RG group (*p* < 0.05), while Arg addition completely reversed these enhancements. The Nrf2 mRNA level was negatively related to the Keap1 mRNA expression (r = −0.9101; *p* < 0.01). The Nrf2 protein expression was negatively related to the Keap1 protein expression (r = −0.9535; *p* < 0.01; Figure 4).

## 4. Discussion

The nutrient levels of feedstuff are closely related to estrus in ewes. Follicular development is a physiological process adapted to direct or indirect nutritional signals. In this study, the estrous cycle days were significantly increased in the RG group compared with the control group, while Arg addition reversed this prolonged estrus in ewes. The weights of the SBW, EBW, ovary, carcass, and the ovary/EBW were not significantly different between the groups. These results mean that there was no significant influence on the body weight during the short-term feeding of the luteal phase, which was in agreement with a previous study [11]. In addition, short-term nutrient restriction resulted in delayed estrus, which was alleviated by short-term Arg addition. Short-term Arg supplementation ameliorated the delayed estrus induced by nutrient restriction, which might be due to the promotion of ovarian follicle development in ewes, which was in agreement with previous studies [11,12]. In addition, nutrient restriction increased the number of primary follicles and inhibited the transformation from primary follicles to secondary follicles [13]. In this study, in the RG group, the number of small follicles (≤2 mm in diameter) was significantly increased and the proportion of large follicles (≥5 mm in diameter) was significantly decreased. Nevertheless, Arg supplementation enhanced the proportion of large follicles (>5 mm in diameter), which might be due to the transformation from primary follicles to secondary follicles induced by the Arg addition. These results mean that Arg stimulates follicular development and increases ovulation, which was in agreement with previous studies [2,14]. Follicular development can be regulated by hormone secretion of the hypothalamic pituitary gonad axis in mammals [15]. In this study, nutrient restriction significantly increased the E2 level in the large follicles (≥5 mm in diameter), while it significantly decreased the E2 level in the small follicles (≤2 mm in diameter). Meanwhile, nutrient restriction significantly decreased the P4 level in the small follicles and the large follicles. These results mean that an excess of P4 in the large follicles might lead to the reduction in large follicles by means of a feedback regulation followed by follicular atresia, resulting in the reduction in large follicles [16]. 

Arg, as a conditionally essential amino acid, is an important substrate for the synthesis of protein [17]. The Arg family of amino acids can be transformed in the body and play important roles in immunity, antioxidants, and so on [17]. In this study, nutrient restriction significantly increased the levels of Glu, Cit, Pro, and Orn, while Arg addition significantly decreased the levels of Pro and Orn, which was in agreement with a previous study [2]. A previous study proved that nutrient restriction significantly increased the levels of Cit, Pro, and Orn by means of inducing oxidative stress in ewes [18]. Nutrition restriction induced oxidative stress by disrupting the mitochondrial energy metabolism and redox dynamic balance [19]. When oxidative stress is triggered, intracellular excess ROS can induce cellular damage and impair the antioxidant capability [5]. Both GSSG and GSH are key antioxidants in intracellular redox regulation, and the activities of SOD, CAT, GSH-Px, and the GSH/GSSG ratio are used as role indicators to evaluate the antioxidant capability in cells [20]. In this study, nutrition restriction significantly increased the MDA level, while it sharply decreased the GSH/GSSG and the activities of SOD, CAT, and GSH-Px. These results mean that nutrition restriction might impair the ovarian antioxidant capability in ewes, which was in agreement a previous study [2]. The properties of SOD, CAT, and GSH-Px were those of proteins and could be regulated by the related genes. In this study, the mRNA expressions of CuZnSOD, CAT, and GSH-Px were consistent with the activities of SOD, CAT, and GSH-Px. Nutrition restriction significantly reduced the mRNA expressions of CuZnSOD, CAT, and GSH-Px, while Arg reversed these reductions, which were in parallel with the activities of the antioxidant enzymes. However, MnSOD mRNA expression was not affected by nutrition restriction in the ovaries. The possible explanation for this mismatched relationship is that there are more than two isoenzymes of SOD, and SOD activity is not equivalent for all the isoenzymes. Nevertheless, the MnSOD enzyme in the ovaries is only one of the isoenzymes, and might not transcript the entire SOD enzyme [21].

There are various signaling pathways to ensure redox homeostasis in cells, including the Nrf2/Keap1 pathway [22]. When the ovarian cells are in a stationary state, Nrf2 binds to Keap1 in the cytoplasm. Nevertheless, excessive intracellular ROS will change the reactive thiol groups of Keap1, inducing the Nrf2 transfer to the nucleus and activating the gene expressions of antioxidant enzymes [23]. In this study, nutrition restriction significantly decreased the expressions of the Nrf2 mRNA and protein, while significantly increased the expressions of the Keap1 mRNA and protein. In addition, positive correlations occurred between the antioxidant enzyme activities and the related mRNA expressions. Meanwhile, the Nrf2 mRNA expression was positively connected with the antioxidant mRNA expressions and negatively related to the Keap1 mRNA expression. Similarly, the Nrf2 protein expression was negatively related to the Keap1 protein expression. This means that the nutrition restriction affected the ovarian antioxidant system, while Arg addition could improve the damage to the ovarian antioxidant capability in ewes, which was related to the Nrf2/Keap1 pathway in this study. Kerasioti et al. (2016) reported that the Nrf2/Keap1 pathway played an important role in the increase in antioxidant activity in sheep muscle cells, which was in agreement with this study [24]. This study suggests that nutrition restriction can reduce the ovarian antioxidant capability in the luteal phase in mammals, which can be significantly improved by Arg supplementation. This process was associated with the Nrf2/Keap1 pathway. 

## 5. Conclusions

**Figure 5:**
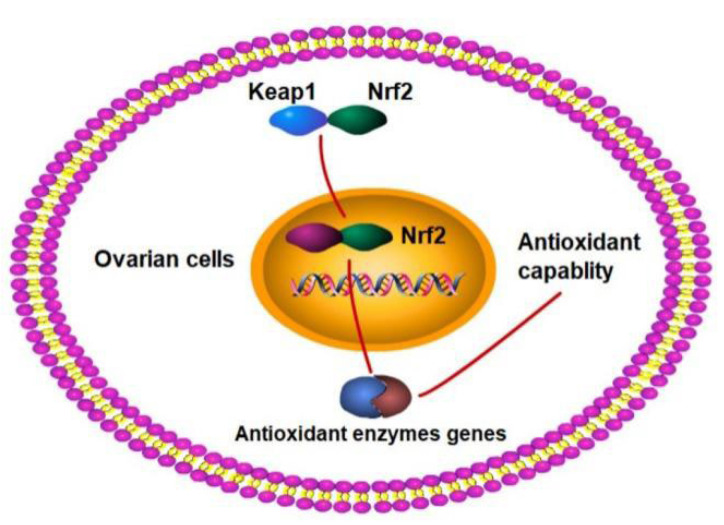
Proposed mechanism of arginine enhancing the ovarian antioxidant capacity during luteal phase in ewes. Nrf2, nuclear factor erythroid 2-related factor 2; Keap1, Kelch-like ECH-associated protein 1.

Nutrition restriction significantly enhanced the number of small follicles (≤2 mm in diameter), increased the levels of Glu, Cit, Pro, and Orn, and reduced the ovarian antioxidant capability in the luteal phase in ewes. However, Arg supplementation enhanced the proportion of large follicles (>5 mm in diameter), decreased the levels of Pro and Orn, and significantly improved the ovarian antioxidant capability. This process was associated with the Nrf2/Keap1 pathway (Figure 5).

## Figures and Tables

**Table 1:** Composition and nutrient content in the basal diet.

Ingredient	g/kg	Nutrient Content	g/kg
Corn	422.50	Dry matter	898.50
Soybean meal	165.00	Crude protein	138.50
Soy straw	400.50	Neutral detergent fiber	487.10
Limestone	3.00	Acid detergent fiber	203.60
Anhydrous calcium phosphate	4.00	Organic matter	908.00
Sodium chloride	4.00	Ash	82.00
Premix ^1^	1.00		

^1^ The premix provided the following nutrients per kg of the diet: 10,000 IU VD, 30,000 IU VA, 100 mg VE, 90 mg Fe, 12.5 mg Cu, 50 mg Mn, 100 mg Zn, 0.3 mg Se, 0.8 mg I, and 0.5 mg Co.

**Table 2:** Sequences of real-time PCR specific primers.

Gene ^1^	Primer	PCR Product (bp)	Accession Number
CuZnSOD	Forward 5′-CACTGCATCATTGGCCGTACCA-3′	223	NM_205064.1
	Reverse 5′-GCTTGCACACGGAAGAGCAAGT-3′		
MnSOD	Forward 5′-CACTCTTCCTGACCTGCCTTAC-3′ Reverse 5′-TAGACGTCCCTGCTCCTTATTA-3′	399	NM_204211.1
GSH-Px	Forward 5′-GCTGTTCGCCTTCCTGAGAG-3′ Reverse 5′-GTTCCAGGAGACGTCGTTGC-3′	118	NM_001277853.1
CAT	Forward 5′-TGGCGGTAGGAGTCTGGTCT-3′ Reverse 5′-GTCCCGTCCGTCAGCCATTT-3′	112	NM_001031215.1
Nrf2	Forward 5′-ATCACCTCTTCTGCACCGAA-3′ Reverse 5′-GCTTTCTCCCGCTCTTTCTG-3′	258	NM_205117.1
Keap1	Forward 5′-TGCCCCTGTGGTCAAAGTG-3′ Reverse 5′-GGTTCGGTTACCGTCCTGC-3′	104	XM_015274015.1
β-actin	Forward 5′-AGCGAACGCCCCCAAAGTTCT-3′ Reverse 5′-AGCTGGGCTGTTGCCTTCACA-3′	139	NM_205518.1

^1^ CuZnSOD, copper-zinc superoxide dismutase; MnSOD, manganese superoxide dismutase; GSH-Px, glutathione peroxidase; CAT, catalase; Nrf2, nuclear factor erythroid 2-related factor 2; Keap1, Kelch-like ECH-associated protein 1.

**Table 3:** Effects of diet and arginine on body weight, ovarian weight, and the number of follicles and corpus luteum in ewes ^1^.

Items	CG	RG	AG	SEM	*p*-Value
SBW, kg	44.35	44.56	44.19	0.435	0.6754
EBW, kg	43.67 ^a^	40.67 ^b^	42.98 ^a^	0.587	0.0432
Ovary, g	1.67	1.58	1.54	0.067	0.1234
Carcass, kg	18.54	19.76	20.43	0.545	0.3234
Ovary/EBW, g/kg	0.038	0.038	0.036	0.003	0.4869
Estrous cycle days	17.54 ^b^	20.21 ^a^	17.43 ^b^	0.434	0.0324
Follicles					
SF	11.23 ^c^	19.43 ^a^	14.32 ^b^	1.456	0.0234
MF	1.21	1.11	1.00	0.543	0.3245
LF	1.54	0.74	1.23	0.342	0.1923
TF	13.98 ^c^	21.28 ^a^	16.55 ^b^	0.432	0.0032
SF/TF	0.80 ^b^	0.91 ^a^	0.87 ^a^	0.323	0.0087
MF/TF	0.08	0.05	0.06	0.435	0.2944
LF/TF	0.11 ^a^	0.03 ^c^	0.07 ^b^	0.576	0.0045
Corpus luteum					
SCL	2.97	3.43	3.23	0.723	0.4832
LCL	0.83 ^b^	0.43 ^b^	1.94 ^a^	0.432	0.0032
TCL	3.80	3.86	5.17	0.868	0.4322
SCL/TCL	0.78	0.89	0.62	0.754	0.2132
LCL/TCL	0.22	0.11	0.38	0.543	0.1765

^1^ CG, control group; RG, restriction group; AG, Arg group; SBW, start body weight; EBW, end body weight; SF, small follicle (≤2 mm in diameter); MF, middle follicle (2–5 mm in diameter); LF, large follicle (≥5 mm in diameter); TF, total follicles = SF + MF + LF; SCL, small corpus luteum (diameter < 5 mm); LCL, large corpus luteum (diameter ≥ 5 mm); TCL, total corpus luteum = SCL + LCL. ^a–c^ Means with different superscript letters differ significantly in the same row (*p* < 0.05). Values are the means ± SE (n = 6).

**Table 4:** Effects of diet and arginine on amino acid metabolism in serum of ewes ^1^.

Items	Treatment			Days					*p*-Value		
	CG	RG	AG	Day 9	Day 10	Day 11	Day 12	Day 13	T	D	T × D
Arg, pg/mL	219.43	220.32	223.21	230.21	220.34	221.32	219.45	215.43	0.81	0.82	0.56
Glu, ng/mL	14.12 ^c^	15.15 ^b^	15.98 ^a^	15.43	15.78	15.65	15.43	16.01	0.02	0.89	0.74
Cit, nmol/mL	63.22 ^b^	79.04 ^a^	78.54 ^a^	75.64	72.12	72.44	75.32	69.43	0.01	0.96	0.78
Pro, ng/mL	123.42 ^c^	150.64 ^a^	140.21 ^b^	133.21	134.31	136.54	139.54	140.32	0.01	0.49	0.93
Orn, pg/mL	120.34 ^b^	140.32 ^a^	102.21 ^c^	125.43	120.43	121.45	117.54	119.54	0.01	0.49	0.02

^1^ Abbreviations: CG, control group; RG, restriction group; AG, Arg group; Arg, Arginine; Glu, glutamic acid; Cit, citrulline; Pro, proline; Orn, ornithine; T, treatment; D, days; T × D, treatment × day interaction. ^a–c^ Means with different superscript letters differ significantly in the same row (*p* < 0.05). Values are the means ± SE (n = 6).

**Table 5:** Correlation coefficients (r) between antioxidant enzymatic activities and antioxidant enzyme gene expressions and between Nrf2 gene expression and expressions of antioxidant enzyme gene or Keap1 gene in ovaries of ewes ^1^.

mRNA Expression Level	Enzymatic Activity	Nrf2 mRNA Expression Level
CuZnSOD	0.843 **	0.794 **
MnSOD	0.209 **	0.183 **
CAT	0.798 **	0.743 **
GSH-Px	0.803 **	0.813 **
Keap1	−0.698 **	−0.783 **

^1^ Abbreviations: CuZnSOD, copper-zinc superoxide dismutase; MnSOD, manganese superoxide dismutase; CAT, catalase; GSH-Px, glutathione peroxidase; Nrf2, nuclear factor erythroid 2-related factor 2. Two asterisk superscripts (**) represent significant at 0.01 level (two-tailed).

## Data Availability

Not applicable.
